# Anaplastic thyroid cancer: Cases, incidence, prevalence and survival in Germany

**DOI:** 10.1007/s12020-026-04643-2

**Published:** 2026-05-04

**Authors:** Susanne Singer, Katherine J. Taylor, Yara Maria Machlah, Tim Brandenburg, Kerstin Lorenz, Sabine Wächter, Klaus Kraywinkel, Markus Luster, Friederike Eilsberger

**Affiliations:** 1 Comprehensive Cancer Center Mecklenburg-Vorpommern (CCC-MV), University Medical Center Rostock, Department of Quality of Life, Rostock, Germany; 2 University Hospital Essen, Department of Endocrinology, Diabetology & Metabolism, Essen, Germany; 3 West German Cancer Center, Essen, Germany; 4 Martin Luther University Halle Wittenberg, Medical Faculty, Department of Visceral, Vascular & Endocrine Surgery, Halle, Saale, Germany; 5 Philipps University Marburg, University Hospital of Giessen & Marburg, Department of Visceral Thorax and Vascular Surgery, Marburg, Germany; 6 German Centre for Cancer Registry Data (ZfKD), Robert Koch Institute, Berlin, Germany; 7 Philipps University Marburg, University Hospital of Giessen & Marburg, Department of Nuclear Medicine, Marburg, Germany

**Keywords:** Thyroid Carcinoma, Anaplastic, Occurrence, Incidence, Risk, Survival, Mortality

## Abstract

**Background:**

Because anaplastic thyroid cancer (ATC) is a rare diagnosis, detailed epidemiological data are difficult to find in publicly accessible platforms. The aim of this study was to report current incidence and survival estimates for ATC in Germany and to analyse their trends over time.

**Methods:**

Data from the population-based cancer registries in Germany were pooled. ATC was classified according to the International Classification of Diseases, 10th edition (ICD-10). Age-standardised incidence rates (per 100,000) for the years 2011 to 2022 were calculated. The relative survival was estimated using period analysis. Annual percentage changes (APC) in the incidence and survival trends over time were computed.

**Findings:**

The number of new ATC cases diagnosed between 2011 and 2022 was 2127 (924 in men and 1203 in women). The current raw incidence is 0.19 in men and 0.22 in women. The age-standardised incidence in men changed slightly, but not significantly, during this period (APC − 2%, *p* = 0.126; recent rate 0.11) while it decreased in women (APC − 3%, *p* < 0.001; recent rate 0.10). The five-year relative survival ranged from 11% (2020–2022) to 15% (2017–2019) and decreased considerably with age. There were no differences in survival between men and women and no evidence for a change in survival over time.

**Conclusion:**

About two persons per 1 million inhabitants are currently diagnosed with ATC per year. Over the past 12 years, there was a slight decrease in ATC incidence, especially in women, while survival remained largely unchanged during this period.

## Introduction

Anaplastic thyroid carcinomas (ATC) are extremely rare, highly aggressive and usually rapidly lethal. The median survival time after diagnosis is approximately a mere three months [1, 2]. For this reason, only tumour stage IV (according to Union Internationale Contre le Cancer, UICC) is assigned to this disease [3]. The tumours commonly exhibit genetic variations that make the cancer exceptionally difficult to treat. However, this characteristic has opened up approaches for immuno-oncological therapies [4–8]. Several years of testing with promising results have led to the incorporation of these therapies into European and American guidelines [9–11].

Because of the rarity of the disease, detailed epidemiological data are difficult to find. Interactive databases of national and international cancer registries (https://www-dep.iarc.fr/today/en) typically do not present their information stratified by histology. In these data sets, ATC cases are usually combined with the other types of thyroid carcinomas, although the incidence and mortality of ATC are distinct. Hence, one has to rely on published articles where ATC specific epidemiological data are presented. The most comprehensive overview so far was provided by a working group from the International Agency for Research on Cancer [12]. They reported age-standardised incidence rates of 0.2 (in Shanghai, Zhongshan, Harbin, and Jiashan, China) to 2 (in Cali, Colombia) per 1 million women and of 0.03 (China) to 1.5 (Switzerland and Austria) per 1 million men. A small but detectable decrease in incidence over time was observed for 21 of the 25 countries investigated. This is in contrast to recent findings from the Surveillance, Epidemiology, and End Results (SEER) program [13], where a slight increase in incidence was found.

It was therefore our aim to investigate the epidemiology of ATC in Germany in more detail. In particular, we aimed to answer the following questions:

1. How has the incidence of ATC in Germany changed over the past years, by age-group and gender?
2. How has the relative survival of ATC in Germany changed over the past years, by age-group and gender?
3. How many people are currently living with ATC in Germany?

We were also interested in the proportion of patients with ATC among all malignant thyroid tumours.

## Methods

### Data source and population

A pooled national data set from the German Centre for Cancer Registry Data (“Zentrum für Krebsregisterdaten”, ZfKD) based at the Robert Koch Institute in Berlin was used. The ZfKD collects data for patients diagnosed with a neoplasm from all 16 federal states in Germany, covering a population of about 84 million inhabitants. All doctors are obliged by law to inform their regional population-based cancer registry about each cancer diagnosis they make. The regional registries collate this information from the various sources, clean the data, and send it to the ZfKD. Because the cancer registration in Germany is regulated by law, no ethical approval was necessary for this study.

We used thyroid cancer morphology codes of the International Statistical Classification of Diseases (ICD) and Related Health Problems, version 10, Code C73, classified according to the ICD for oncology (ICD-O-3) histology for anaplastic thyroid cancer (8020 and 8021). Only patients ≥ 18 years of age at diagnosis were included. Data export for the calendar years 2011 to 2022 was performed in February and March 2025 (with 2022 being the most recent year for which data were available^1^).

### Analysis

Incidence was estimated as number of cases per 100,000 inhabitants, age-standardised by the old European standard population and per gender, per age group, and per calendar year. Because of the rarity of the disease, age and calendar year were aggregated to larger groups in order to reduce the risk of re-identification and to avoid spurious results.

Relative survival was estimated using period analysis [14, 15]. This approach has been shown to provide more up-to-date survival estimates than traditional cohort analysis. In period analysis, survival analysis is limited to a specific calendar period by truncating survival times at the beginning of the period of interest on the left and censoring them at the right end. Period analysis has been found to accurately predict the subsequently observed survival rate of patients diagnosed during the period of interest. The relative survival was calculated as the ratio of the observed survival in the group of ATC patients to the expected survival in a general population comparable in gender and age. The expected survival was derived using the Ederer II method and German life tables from 1993 to 2022 provided by the Federal Statistical Office [16]. For the survival analyses, only registries with sufficient data quality regarding vital status were included.

Annual percentage changes (APC) in the trend of incidence and five-year survival were calculated with the Joinpoint software of the National Cancer Institute (Surveillance Research Program, National Cancer Institute, Joinpoint Regression Software, Version 5.4.0 - April 2025). The slopes of the regression lines were tested to see if they were significantly different from zero.

Age- and gender-specific partial prevalence estimates were calculated for one, three, and five years. Partial prevalence was defined as the proportion of people still living with ATC at the end of a given year (on December 31) who were diagnosed in the previous one, three, or five years [17].

## Results

### Case numbers

The total number of new ATC cases diagnosed between 2011 and 2022 was 2127 (924 in men and 1203 in women). That means, per year on average, 177 individuals in Germany were newly diagnosed with ATC during this 12-year period (provided all cases were included in the cancer registries).

The proportion of these cases among all thyroid cancer diagnoses varied by age. Among patients 20 to 39 years old, they accounted for 0.2% in men and 0.1% in women on average. In the age group 40 to 59 years, the proportion was 2% and 0.6% respectively; age group 60 to 79: 7% and 5%. Among patients 80 years and older, 16% of all thyroid cancers in men and 15% in women were ATC. Overall, 4% of all thyroid cancers in men and 2% in women are ATC.

### Incidence

**Fig. 1:**
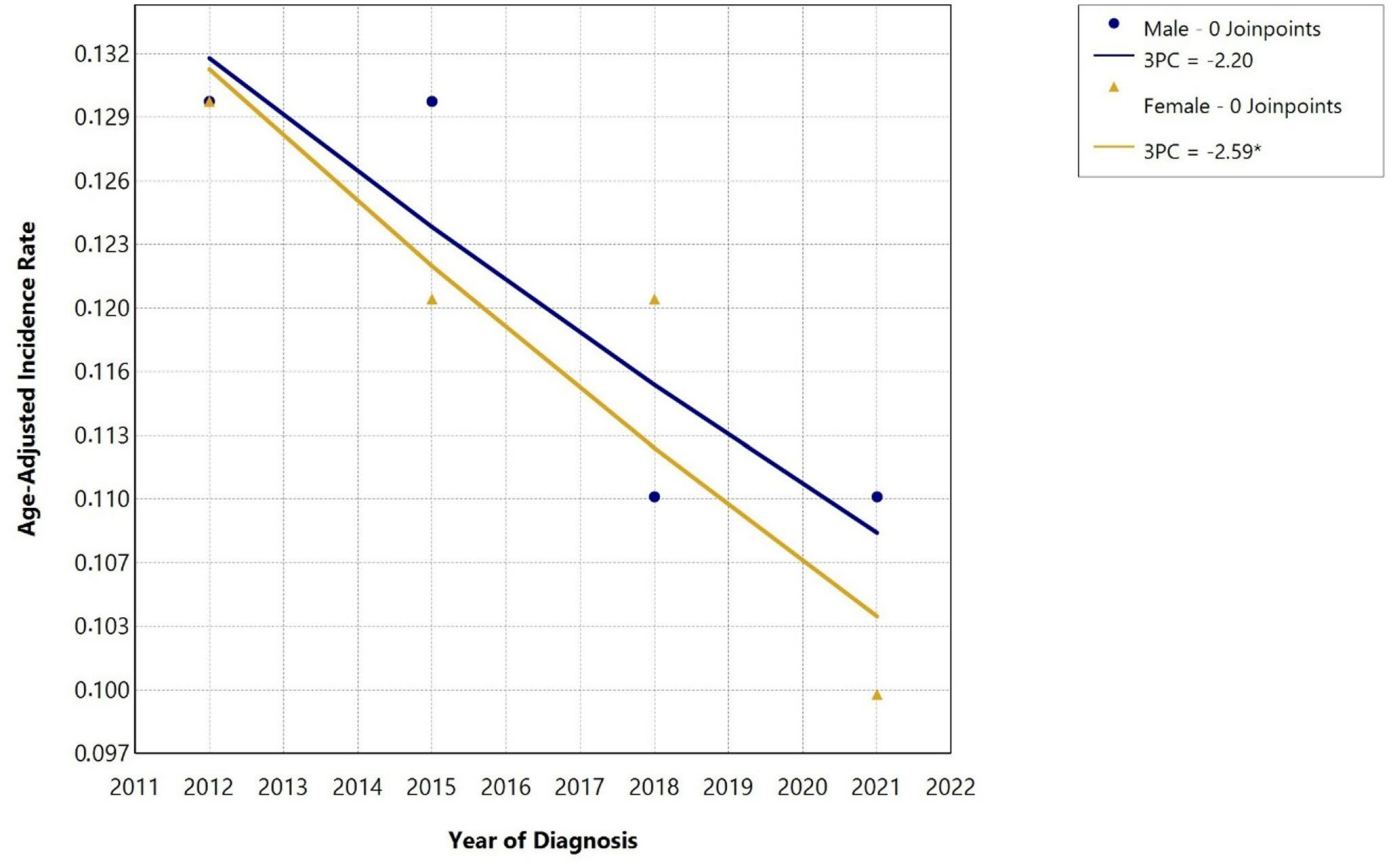
Trends in age-adjusted anaplastic thyroid carcinoma incidence in men and women in Germany, 2011–2022. Note: Incidence estimates were aggregated into 3-year bands. The estimates are displayed at the middle year of each band. The * indicates statistically significant changes

The raw incidence per 100,000 for the most recent period (2020–2022) was 0.19 in men and 0.22 in women. The age-standardised incidence per 100,000 for men and women is shown in Table 1 and the trend over time in Fig. 1. The incidence in men did not change significantly during this period (APC − 2%, 95% Confidence Interval [CI] -4.8 to 0.5, *p* = 0.126) while it decreased slightly in women (APC − 3%, 95% CI -4.1 to -1.0, *p* < 0.001).

**Table 1:** Age-standardised incidence of anaplastic thyroid cancer (per 100,000)

Gender	Age in years	2011–2013	2014–2016	2017–2019	2020–2022
Male	20–39	0.00	0.01	0.01	0.01
	40–59	0.09	0.12	0.10	0.08
	60–79	0.59	0.56	0.43	0.52
	80+	0.99	0.86	0.87	0.81
	*All males*	*0.13*	*0.13*	*0.11*	*0.11*
Female	20–39	0.01	0.01	0.00	0.01
	40–59	0.10	0.10	0.09	0.06
	60–79	0.51	0.47	0.52	0.49
	80+	1.42	1.21	1.12	0.88
	*All females*	*0.13*	*0.12*	*0.12*	*0.10*

The highest age-standardised incidence was found in women 80 years and older, with a peak of 1.4 new cases per 100,000 women in the period 2011–2013, whereas in the same period no men under the age of 40 were diagnosed with ATC.

### Survival

**Fig. 2:**
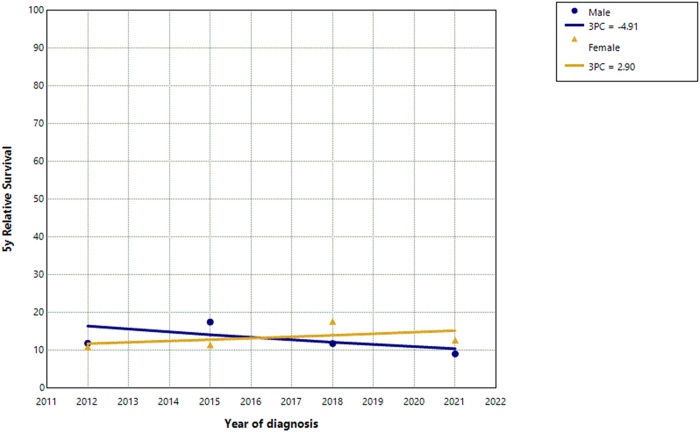
Trends in 5 year relative survival in patients with anaplastic thyroid carcinoma in men and women in Germany, 2011–2022. Note: Survival estimates were aggregated into 3-year bands. The estimates are displayed at the middle year of each band

**Fig. 3:**
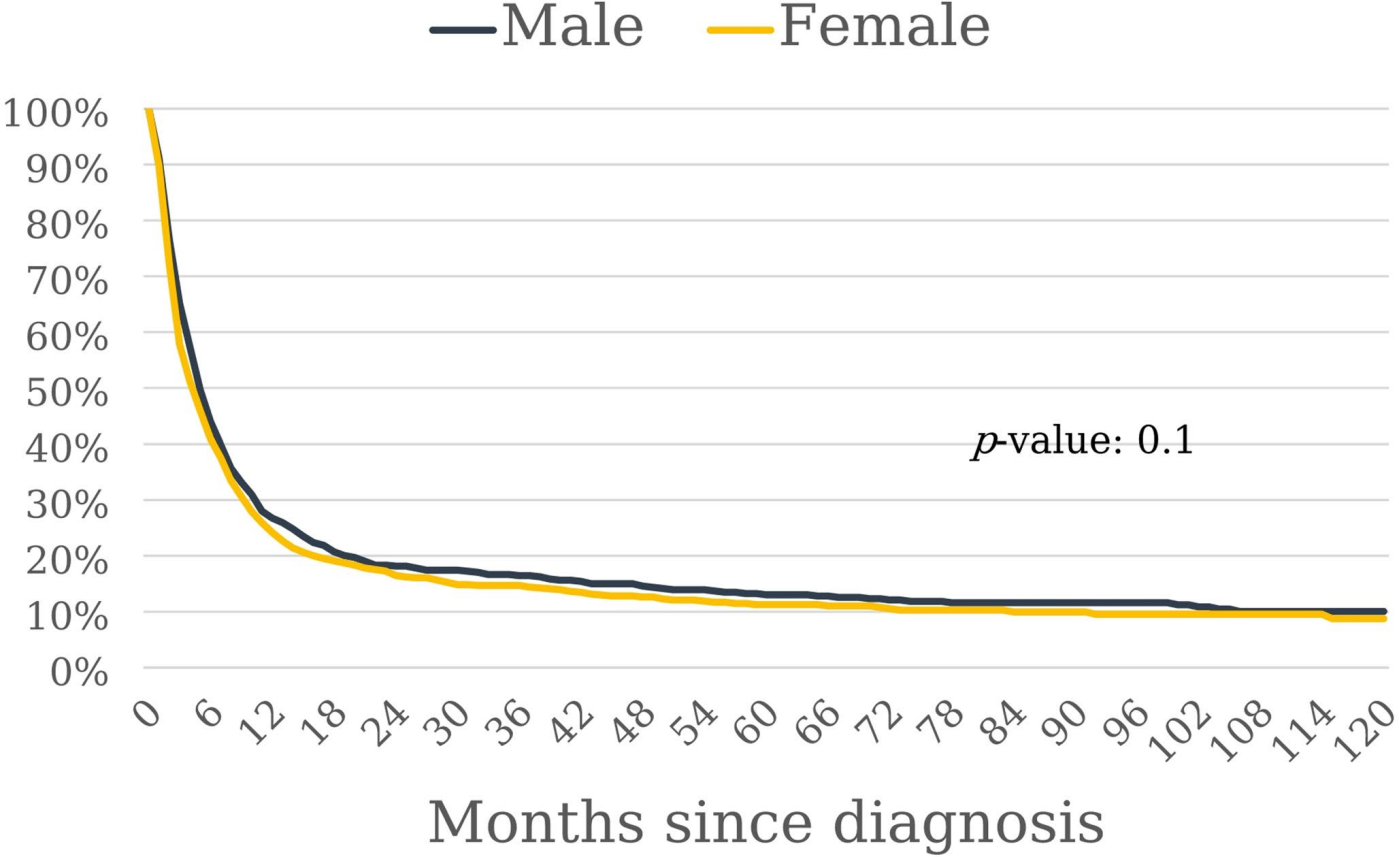
Percentage of patients with anaplastic thyroid carcinoma surviving after diagnosis. Note: The graph is based on data from 681 men and 864 women in Germany. Log-rank test: χ^2^ = 2.7, *p* = 0.1

The total number of deaths associated with ATC between 2011 and 2022 was 1637 (723 in men and 914 in women). The five-year relative survival after diagnosis ranged from 11% in the most recent period to 15% in 2017–2019 and decreased considerably with age (Table 2). There were no differences in survival between men and women and no evidence for a change in survival over time (Figs. 2 and 3). The APC was − 4.9 in men (95% CI -16.5 to 6.7, *p* = 0.354) and 2.9 in women (95% CI -4.4 to 11.8, *p* = 0.398). The log rank test for differences in survival between men and women revealed χ^2^ = 2.7 and *p* = 0.100.

**Table 2:** Five-year relative survival after diagnosis of anaplastic thyroid cancer, by calendar year, age, and gender

Age in years and gender	# cases	2011–2013	2014–2016	2017–2019	2020–2022	2011–2022
18–39	10					32.4%
40–59	299	21.4%	29.3%	21.4%	24.5%	
60–79	892	12.2%	12.6%	15.3%	11.7%	
80+	427	4.2%	8.4%	13.2%	2.9%	
Male	721	11.9%	17.5%	11.8%	9.1%	
Female	914	10.9%	11.5%	17.7%	12.7%	
*Overall*		*11.6%*	*14.5%*	*14.9%*	*11.3%*	

Note: Due to low case numbers, the survival data in 18–39 year old patients was aggregated over all time periods

The one to ten-year relative survival estimates for the most recent period (2020–2022) are shown in Table 3. In all strata, older patients had lower survival than younger ones.

**Table 3:** One to ten year relative survival after diagnosis of anaplastic thyroid carcinoma in the period 2020–2022, by age and gender

Time since diagnosis	40–59 years	60–79 years	80 + years	18 + years	Men	Women
*N*	*77*	*255*	*119*	*456*	*203*	*254*
1 year	39.9%	29.4%	12.2%	26.2%	29.9%	23.5%
2 year	28.3%	21.2%	8.1%	18.5%	16.9%	19.1%
3 year	28.4%	18.5%	4.8%	15.9%	13.8%	16.9%
4 year	26.5%	15.8%	3.0%	13.5%	12.0%	14.2%
5 year	24.5%	11.7%	2.9%	11.3%	9.1%	12.7%
6 year	24.7%	10.1%	3.5%	10.8%	8.1%	12.7%
7 year	24.9%	10.5%	3.4%	11.0%	8.5%	12.7%
8 year	25.1%	11.0%	4.3%	11.6%	8.9%	13.5%
9 year	25.3%	10.8%	5.7%	11.8%	8.8%	14.4%
10 year	25.5%	11.5%	5.3%	12.1%	9.2%	14.2%

### Prevalence

The estimated one-year, three-year, and five-year prevalence of ATC per 100,000 inhabitants in the different age groups and per gender are shown in Table 4.

**Table 4:** Prevalence of anaplastic thyroid carcinoma (per 100,000)

Gender	Age group	1 year prevalence	3 year prevalence	5 year prevalence
Male	20–39		0.01	0.02
	40–59	0.03	0.11	0.16
	60–79	0.27	0.52	0.60
	80+	0.38	0.55	0.55
Female	20–39	0.02	0.02	0.03
	40–59	0.04	0.10	0.17
	60–79	0.32	0.60	0.82
	80+	0.11	0.27	0.43

## Discussion

This study analysed the recent incidence and survival trends of ATC in Germany. About 2 individuals per 1 million inhabitants are currently diagnosed with ATC per year. This proportion is similar for men and women, in contrast to other thyroid carcinomas which occur more often in women [12]. However, in ATC, this equal male-to-female ratio is a common pattern and was found by Aschebrook-Kilfoy and her team among 393 ATC patients with white ethnicity in the United States [18]. In our data set, the proportion of ATC of all thyroid carcinomas was 4% in men and 2% in women, similar to other countries [12].

In contrast to findings from Northern America [13] and the Netherlands [19, 20] but in concordance with data from other countries [12], we found a slight decrease in age-standardised ATC incidence, especially in women. This decline is difficult to explain. One idea would be that overall, the exact histopathological classification has improved, as has the understanding that ATC “prevails” over other entities whenever heterogeneous tumours are present [21]. However, this does not explain any gender-specific differences. Another explanation is the decline in ATC incidence following iodine supplementation, most likely due to fewer cases of nodular goitre with possible follicular thyroid carcinomas, which then dedifferentiate into ATC [22]. Early detection and treatment of small tumours might prevent cases from developing into ATC [23] and it is possible that this happens more often in women as they tend to use health care services and attend check-ups more often than men [24–26], they also received more often thyroid nodule ultrasound due to palpable neck mass in an Italian study [27], but in another analysis from Portugal, no sex differences in the proportion of incidental versus non-incidental diagnoses of papillary thyroid carcinomas were found [28].

There was no statistical evidence for a change in survival. This came as a surprise as new treatment options have emerged in recent years [3] and one would have hoped to see their effects in the survival curves. One explanation for this could be the aggregation of data into 3 year bands, which was necessary to avoid spurious findings but might have blurred a potential benefit of the new treatment strategies. This assumption is backed up by the fact that the most recent one year survival of 26% was higher than in previous years, where it was well below 20% [29, 30]. As well, findings by colleagues at the University of Texas MD Anderson Cancer Center show that it is no longer appropriate to view ATC patients with the hopelessness that characterised treatment in previous decades [31, 32]. Another explanation for why increased survival is not yet evident in the data is that not all hospitals have adopted the new treatment strategies, especially if they do not specialise in treating ATC.

Furthermore, unlike in the United States, no targeted therapies or immunotherapies for the treatment of ATC are currently approved in Europe, including BRAF inhibitors and immune checkpoint inhibitors. In Europe, these are only available for off-label use. This regulatory difference may have contributed to the fact that no improved survival was observed in the population studied. Further epidemiological studies should investigate this in more detail by combining treatment information and molecular profiles with survival data.

Our study underlines once more the importance of age in regard to incidence. In contrast to other thyroid carcinomas, ATC appear most often in older ages [12]. This makes treatment even more difficult, as older patients often suffer from co-morbid diseases and may have a poor health status [33], which must be considered during treatment planning [34]. Moreover, access to health care, diagnostic and especially expensive treatment modalities sometimes are impeded or even denied for older patients.

A limitation of this study is that not all of the federal states had sufficiently complete data so that the incidence had to be estimated and could not be counted. This situation has improved in recent years because of better funding for cancer registration in Germany, so future data analysis might be even more precise. Another limitation, though unavoidable due to the rarity of the disease, is the small sample size, especially in the younger age groups, which therefore had to be combined into 3-year periods. We retained this approach because reporting age-specific incidence and survival is important, as both epidemiological estimates differ considerably across age groups.

In summary, our study reports current epidemiological estimates for ATC in Germany. Our findings indicate that ATC incidence has decreased slightly, potentially due to better detection of early tumours. The most recent survival findings are in line with the assumption that improved treatment is changing survival, but the evidence is limited. Future community-based studies should combine treatment data with outcomes to investigate this further. Moreover, larger data sets combining survival data from different countries might be needed. Our results can be used as a basis for a meta-analysis in this respect.

## Data Availability

The data used in this study are fully available at the Centre for Cancer Registry Data (ZfKD), Berlin, Germany. To that end a justified scientific interest must be credibly demonstrated. On approval of the application following assessment by the Scientific Board of the ZfKD, the anonymised data is made available for the respective planned project.

## Footnotes

1 There is a time lag between the date when doctors inform the cancer registry and the date the cancer registry sends this data to the ZfKD because the registry need to check the data quality and collate various sources of information. They then prepare a “best-of” dataset which contains the best available information per patient from any source. This again takes time, so the date when the ZfKD receives the “best-of-data” for a given year used to be up to two or three years after the actual event (diagnosis or death) happened. This has changed recently to one year because of new regulations. The ZfKD then needs about 3 to 6 months for data checking, queries etc., before the data set is available for third parties.
